# The association between remoteness of injury and in-hospital mortality for motor vehicle collision major trauma patients: evidence of survivor bias in an analysis of registry data

**DOI:** 10.1186/s40621-025-00586-w

**Published:** 2025-07-08

**Authors:** David J Read, Ian Hayes, Sheena G. Sullivan

**Affiliations:** 1 Department of Surgery, University of Melbourne, Melbourne, Australia; 2 Trauma Service, Royal Melbourne Hospital, Melbourne, Australia; 3 School of Population and Global Health, University of Melbourne, Melbourne, Australia; 4 WHO Collaborating Centre for Reference and Research on Influenza, Royal Melbourne Hospital, Melbourne, Australia

**Keywords:** Injury, Major trauma, In-hospital mortality, Remoteness, Rurality, Road traffic injury, Motor vehicle accident

## Abstract

**Background:**

Rural Australians have a higher age adjusted rate of both hospitalisation and death from injury, and this rate increases with increasing remoteness. However, it is uncertain if this is due to an increased incidence of injury or less access to treatment. The aim of this study is to examine the association of remoteness and in-hospital mortality in Major Trauma patients admitted to the Royal Melbourne Hospital.

**Methods:**

This study was a retrospective cohort study of all persons aged 15 + years diagnosed with ‘major trauma’, (defined as Injury Severity Score, ISS > 12) from a Motor Vehicle Collision admitted to the Royal Melbourne Hospital from 2010 to 2021. The exposure of interest was remoteness as measured by the Accessibility/Remoteness Index of Australia (ARIA), the outcome of interest was in-hospital mortality. Logistic regression models were constructed looking at the odds of death by increasing remoteness adjusting for age, ISS, and comorbidity. Missing data were imputed using chained equations. A sensitivity analysis was performed for ARIA+ category, and a quantitative bias analysis performed for potential selection bias. All analyses were performed using Stata v17. Ethical approval was obtained from the Melbourne Health Human Research Ethics Committee (HREC2022_044).

**Results:**

Eligibility was met for 2324 cases, of whom 53.3% were classified as major city, 36.1% inner regional, and 10.6% outer regional/remote. In-hospital mortality was 6.0% for those injured in major cities, 5.4% in inner regional and 4.1% for outer regional/remote. The median ISS was 19 and 18.3% had at least one limiting comorbidity. The adjusted odds of death were reduced by half for those injured in outer regional and remote compared with major city (OR = 0.51, 95%CI = 0.25–1.03). This result did not alter with the sensitivity analysis for postcode of injury. Quantitative bias analysis suggested the presence of severe selection bias, with the odds ratio showing an increased odds of death (OR = 1.83) for more remotely injured persons.

**Conclusion:**

Persons injured remotely are not more likely to die in-hospital after major trauma once they arrive at hospital. Unexpectedly, there was some evidence to suggest that those injured most remotely had a survival advantage, despite similar injury severity Quantitative bias analysis suggested selection bias could be responsible for this apparent survival advantage for more remotely injured persons.

**Supplementary Information:**

The online version contains supplementary material available at 10.1186/s40621-025-00586-w.

## Introduction

In Australia, injury is responsible for over 13,000 deaths per year and the leading cause of death for Australians aged 1–44 years [1]. It is well established that regional-living Australians have a higher age-adjusted rate of both hospitalisation and death from injury, and this rate increases with increasing remoteness [2]. Studies from Western Australia [3], the Northern Territory [4] and pooled Australian data [5] have all shown increasing odds of death from injury as remoteness of injury increases. This association has also been observed in motor vehicle crash victims in the USA [6, 7]. It is uncertain what proportion of this difference is due to increased opportunity for injury in remote areas [8], reduced access to timely trauma care [2] and described differences in comorbidity [2]. The Victoria State Trauma System (VSTS) guidelines recommend that all patients with suspected ‘major trauma’ should be managed at a Major Trauma Centre, irrespective of remoteness of injury. Ideally, mortality should be equivalent once the major trauma patient reaches definitive care at the major trauma centre. The aim of this study is to examine the association between remoteness of injury and in-hospital mortality at a major trauma centre in Melbourne, Australia. We hypothesised that increasing remoteness of injury would be associated with increasing in-hospital mortality.

## Methods

### Setting & participants

We constructed a retrospective cohort of patients admitted to the Royal Melbourne Hospital (RMH) for motor-vehicle related major trauma to assess the relationship between remoteness of injury and risk of death. The RMH is one of two Level One Major Trauma Centres in the State of Victoria caring for over 1,000 major trauma patients per year. The RMH receives patients from all over Victoria plus southern New South Wales (NSW). The details of all trauma patients are collated in the RMH Trauma Registry (RMH-TR), an in-house database curated by qualified health information officers. Data recorded include demographic variables (date of birth, sex, postcode of residence), injury information (mechanism, type, severity score), hospitalisation, postcode of residence and injury.

Patients aged 15 and above admitted for major trauma associated with a motor vehicle crash between 2010 and 2021 were included. Major trauma was defined as Injury Severity Score (ISS) > 12. Patients were excluded if they were repatriations from areas outside the RMH catchment or interhospital transfers > 48 h after admission to the transferring hospital. Motorcyclist and pedestrians were also excluded because they are considered vulnerable road users and have disproportionate risk of death, differing injury patterns and severity [9].

**Fig. 1:**
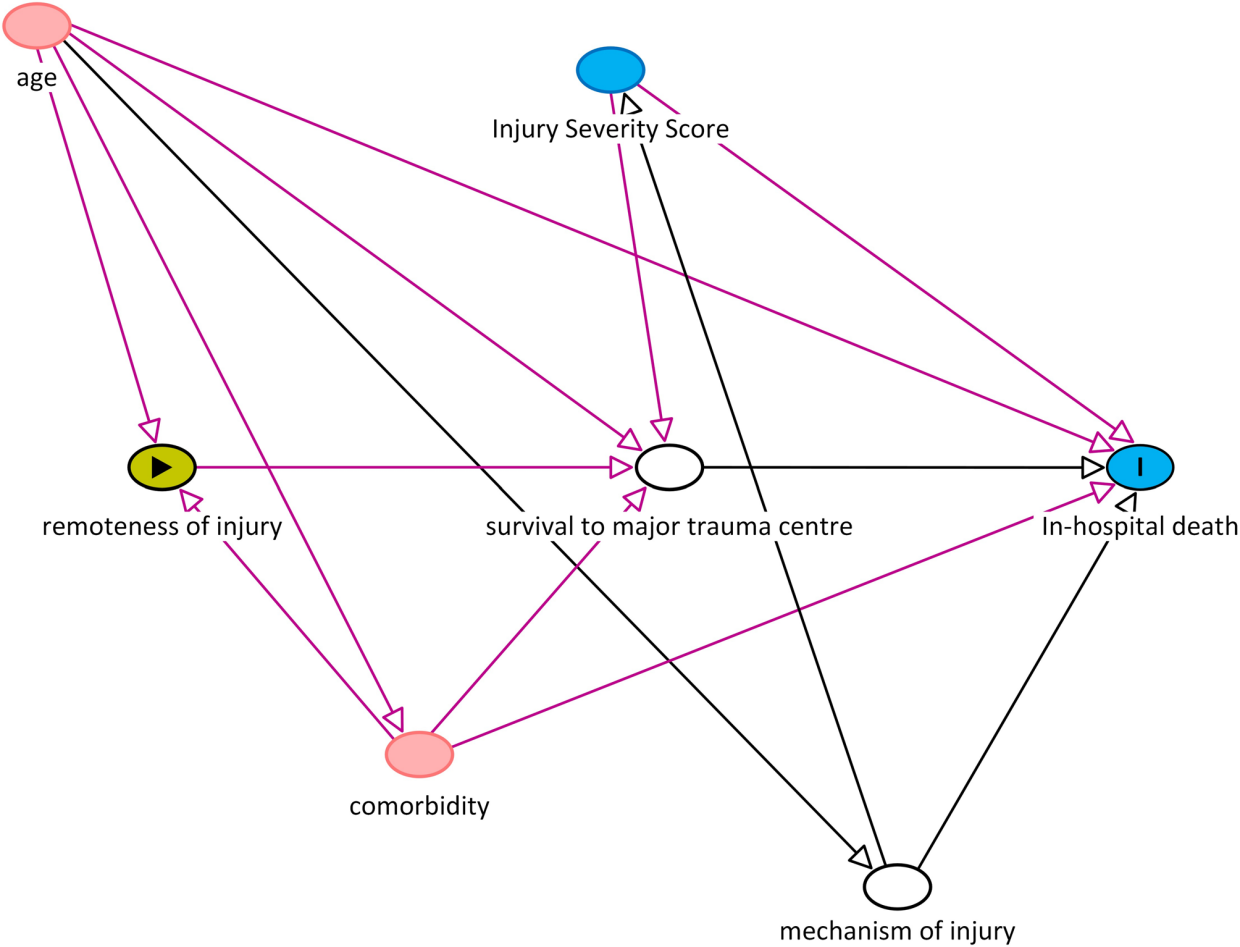
Directed acyclic graph showing the assumed causal model for the main analysis

The causal relationship between remoteness of injury and in-hospital death was guided by the directed acyclic graph shown in Fig. 1. The main outcome of interest was in-hospital mortality, as recorded in the RMH-TR. This included death whilst an inpatient at the RMH, but not if death occurred after discharge home or to a rehabilitation facility. Remoteness of injury was measured by Accessibility/Remoteness Index of Australia Plus (ARIA+) category a measure of access to differing levels of service by road, and estimable from postcode. The five-tiered ARIA+ categorisation was contracted to three, namely (1) major cities; (2) inner regional; and (3) outer regional and remote, as there are no “very remote” and very few “remote” locations in Victoria [10]. If the postcode of injury was missing, ARIA+ was imputed based on postcode of residence. Where more than one ARIA+ was associated with a post-code, the ARIA+ category that matched a majority of the population in that post-code was used.

### Data analysis

Descriptive statistics were used to describe sample characteristics by ARIA+ category, using counts and percentages for categorical variables and means with standard deviation for continuous variables. For the main analysis, a logistic regression model was constructed with in-hospital death as the outcome and ARIA+ category as the main exposure, controlling for age group (15–64, 65 + years), ISS category (high > 24 versus moderate 13–24) and comorbidity (healthy/non-limiting versus limiting/constant threat to life). Potential effect modification by age group and ISS on the relationship between ARIA+ category and mortality were explored with the use of interaction terms.

### Sensitivity analyses

Eight-one cases had missing comorbidity data, which was explored by cross tabulation and comparing summary measures of potential reasons for this discrepancy. Missing comorbidity status was imputed using multiple imputation [10] by chained equations (MICE) with age, ISS, death, gender and ARIA+ category as predictors. The logistic regression model was then rerun using the imputed datasets for comparison with the main model to assess the potential bias associated with missing comorbidity data.

Sixty-seven of the 674 postcodes in Victoria cover two or three ARIA+ categories. In the main analysis the majority ARIA+ category for postcodes with multiple ARIA+ categories was selected. In sensitivity analysis, the second most common ARIA+ category was used. Only one postcode had three ARIA+ categories.

### Quantitative bias analysis

Because patients who incur injuries in more remote areas may have a higher risk of death prior to reaching a hospital, a quantitative bias analysis was performed to evaluate potential selection bias due to participation in our study conditional upon survival to a major trauma centre. Quantitative Bias Analysis (QBA) [11] is a tool that estimates both the potential magnitude and direction of bias present in observational studies. When examining selection bias, it requires estimates of the proportion of participants that suffered the outcome for each of the categories of the exposure, that were or were not included in the study, which is referred to as the ‘participation fraction’. In this study we used the proportion of persons with major trauma from a motor vehicle crash, that died or survived, for each stratum of remoteness, that did not make it to a major trauma centre. In this way, an estimate of the potential existence of ‘survival bias’ i.e. more remotely injured persons having a superior survival because the more severely remotely injured have died prior to reaching the major trauma centre. The estimate from a QBA is a number similar to an odds ratio, and can take on a negative value, indicating the direction of the association has changed. In our analysis we performed a deterministic QBA and hence do not report confidence intervals [11]. Participation fractions were based on the proportion of injured persons aged ≥ 16 years involved in a motor vehicle accident with ISS > 12 who survived to admission to a major trauma centre throughout Victoria, which were obtained from the Victorian State Trauma Outcomes Registry (VSTORM) [12]. This registry receives regular updates from the coronial system to ensure data completeness for prehospital deaths. For this analysis we assumed that: (1) the fraction of patients arriving alive at the RMH was comparable with other major trauma centres, noting the similar survival metrics and caseloads in published reports [9]; (2) participation fractions were constant over the study period; and (3) patients who died in the prehospital phase had an ISS > 12 and would therefore have met inclusion criterion had they survived. Bias was assessed based on a deterministic approach using the episens command in Stata [13]. Two models were constructed based on differing cut-off points for this study’s three tier ARIA+ categorisation, firstly major city versus inner/outer regional and remote, then a second model with major city & inner regional versus outer regional & remote.

## Results

**Fig. 2:**
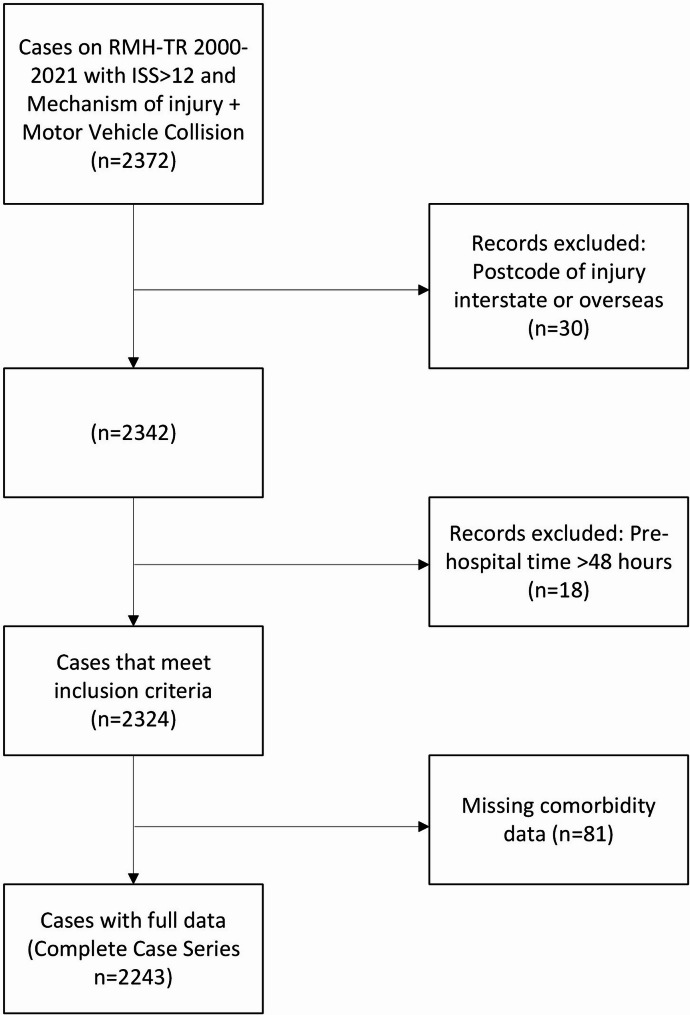
Case acquisition

After data cleaning, 2,324 patients met inclusion criteria (Fig. 2). Mean age was 44.3 (+/- 21.2) years and 62.2% were male. Median ISS was 19 (IQR 14–27) and 26.3% suffered a major head injury. Comorbidity was limiting or a constant threat to life in 18.3%, and 18.8% of cases were an inter-hospital transfer. By ARIA+ category 1,239 (53.3%) were categorised as sustaining an injury in major cities, 838 (36.1%) inner regional and 247 (10.6%) outer regional or remote. Demographics, comorbidity classification, ISS, mortality and prehospital transport factors, stratified by ARIA+ remoteness categories, are presented in Table 1. Age, gender and comorbidities were similar across remoteness categories. Median ISS (IQR) increased with remoteness and was 17 [12] for major cities, 20 [12] for inner regional and 22 [12] for outer regional/remote. In-hospital mortality decreased with remoteness and was 6.0% for major cities, 5.4% for inner regional and 4.1% for outer regional/remote regions. Pre-hospital time and chance of aeromedical evacuation both increased with remoteness.

**Table 1:** Sample description of variable by ARIA+ category

		Major Cities	Inner Regional	Outer Regional/Remote
n	n (%)	1239 (53.3)	838 (36.1)	247 (10.6)
Age	Mean (sd)	44.8 (21.7)	44.1 (20.4)	42.8 (21.7)
	> 65 n (%)	289 (23.3)	170 (20.3)	50 (20.2)
Gender	Male n (%)	774 (62.7)	509 (60.7)	163 (66.0)
Comorbidity	Nil n (%)	396 (32.0)	259 (30.9)	77 (31.2)
	Non limiting n (%)	582 (47.0)	399 (47.6)	119 (48.2)
	Limiting n (%)	213 (17.2)	141 (16.8)	44 (17.8)
	Constant threat n (%)	8 (0.7)	5 (0.6)	0 (0.0)
	Missing n (%)	40 (3.1)	34 (4.1)	7 (2.8)
Injury characteristics & outcome	ISS median (IQR)	17 (12)	20 (12)	22(12)
	ISS 13–24, n (%)	909 (73.4)	581 (69.3)	159 (64.4)
	ISS 25–75 n (%)	330 (26.6)	257 (30.7)	88 (35.6)
	Major head injury n (%)	318 (25.7)	235 (28.0)	63 (25.5)
	Death n (%)	74 (6.0)	45 (5.4)	10 (4.1)
Prehospital transport	Pre-hospital time < 1 h n (%)	187 (15.1)	6 (0.7)	0 (0)
	1–6 h n (%)	930 (75.1)	543 (64.8)	120 (48.6)
	6–24 h n (%)	122 (9.8)	289 (34.5)	127 (51.4)
	Road n (%)	1045 (84.3)	337 (40.2)	63 (25.5)
	Aeromedical evacuation n (%)	132 (10.7)	402 (48.0)	147 (59.5)
	Unknown n (%)	62 (5.0)	99 (11.8)	37 (15.0)

Data were complete for the outcome of interest (death), and mostly complete for the exposure of interest (remoteness). Postcode was imputed from the postcode of residence, rather than injury, for 18 of 2324 patients. The correlation coefficient for ARIA+ category for injury postcode and home postcode was 0.70. Key covariates, ISS and age, were complete, but 81 patients were missing comorbidity data. Missingness was differential with respect to the outcome, death with only 3.8% mortality among those with comorbid conditions listed, but 54.3% among the 81 missing comorbidities. In-hospital length of stay was lower in the group missing comorbidity status (median 1.4 days versus 7.7 days among those with complete data) (Supplementary Table 1). Half (51.9%) of those with missing comorbidity data had a severe head injury compared with 25.6% of those with comorbidity data, and 70.4% of those with missing comorbidity data had an ISS > 25 versus 27.6% of those with complete data. There was no difference in missing data by ARIA+ category, but those with complete data were more likely (19.3% v 6.2%) to have been transferred from another hospital.

Data were analysed by logistic regression, using both complete case analysis (2243 of 2324 cases) and the imputed datasets, to examine the association between remoteness of injury and the odds of death, controlling for age, ISS and comorbidity status (Table 2). We also considered the interaction between age group and ISS but as it was not statistically significant we did not include it in the final model. In the complete case analysis, the odds of death were lower but statistically insignificant in both inner regional (OR = 0.85; 95% CI 0.51–1.39; *p* = 0.50) and outer regional/remote categories (OR = 0.65; 95% CI: 0.29–1.45; *p* = 0.29) compared to major cities.

**Table 2:** Logistic regression for odds of in-hospital mortality by remoteness of injury, adjusting for age, ISS & comorbidity, complete case analysis and imputed datasets

	Complete Case Analysis n = 2243			Imputed Data Analysis n = 2324		
	OR	95% CI	*p*	OR	95% CI	*p*
Remoteness						
Major city	ref			ref		
Inner Regional	0.85	0.51–1.39	0.508	0.81	0.54–1.22	0.323
Outer Regional & Remote	0.65	0.29–1.45	0.291	0.51	0.25–1.03	0.062
Comorbidity						
None or non-limiting	ref			ref		
Limiting or constant threat	2.17	1.33–3.53	0.002	2.01	1.21–3.33	0.007
ISS						
12–24	ref			ref		
25–76	9.36	5.63–15.56	< 0.001	11.01	7.13–17.00	< 0.001
Gender						
Female	ref			ref		
Male	1.35	0.82–2.20	0.240	1.12	0.75–1.68	0.569
Age						
15-64y	ref			ref		
65y+	5.08	3.16–8.17	< 0.001	3.53	2.35–5.30	< 0.001

OR: odds ratio; 95%CI: 95% confidence interval; ref: reference level used in the regression

The complete case analysis has complete data for the 2243 cases

The imputed dataset represents the most complete data, *n* = 2324, with missing comorbidity data imputed using chained equations

The strongest predictor of death was ISS > 25 (OR = 9.57; 95% CI: 5.8–15.9; *p* = 0.000), with age > 65 (OR = 4.86; 95%CI: 3.0–7.7; *p* = 0.000), and presence of a comorbidity (OR = 2.17; 95%CI: 1.3–3.5; *p* = 0.002) also strong predictors of death.

When the analysis was repeated using the imputed datasets, the odds of death for the inner regional compared with major city were comparable with the complete case analysis (OR = 0.81; 95%CI: 0.54–1.22; *p* = 0.32) (Table 2). However, the odds ratio for the outer regional/remote category attenuated from 0.65 to 0.51 (95%CI: 0.25–1.03; *p* = 0.062). The remaining covariates maintained their similar strong association with mortality, with ISS > 25 (OR = 11.01; 95%CI: 7.13–17.0), age > 65 years (OR 3.53, 95%CI: 2.35–5.30) and comorbidity (OR = 2.01; 95%CI: 1.21–3.33).

A sensitivity analysis for ARIA+ category in postcodes with > 1 category was performed using the imputed datasets. This resulted in a reclassification of ARIA+ category for approximately 15% of postcodes, affecting 391 of 2324 (16.8%) of records. Table 3 shows that results were comparable with the original analysis for outer regional/remote (OR = 0.52; 95%CI: 0.27–0.99; *p* = 0.045) but closer to the null for inner regional (OR = 0.97 95%CI: 0.65–1.46) (Table 3).

**Table 3:** Sensitivity analysis for odds of in-hospital mortality by remoteness of injury, adjusting for age, ISS & comorbidity, imputed dataset

	Complete Case Analysis n = 2243			Imputed Data Analysis n = 2324		
	OR	95% CI	*p*	OR	95% CI	*P*
Remoteness						
Major city	ref			ref		
Inner Regional	0.81	0.54–1.22	0.323	0.97	0.65–1.46	0.916
Outer Regional & Remote	0.51	0.25–1.03	0.062	0.52	0.27–0.99	0.045
Comorbidity						
None or non-limiting	ref			ref		
Limiting or constant threat	2.01	0.21–3.33	0.007	2.00	1.23–3.24	0.005
ISS						
12–24	ref			ref		
25–76	11.01	7.13–17.0	< 0.001	10.95	7.08–16.91	< 0.001
Gender						
Female	ref			ref		
Male	1.12	0.75–1.68	0.569	1.12	0.75–1.68	0.55
Age						
15-64y	ref			ref		
65y+	3.53	2.35–5.30	< 0.001	3.52	2.34–5.28	< 0.001

OR: odds ratio; 95%CI: 95% confidence interval; ref: reference level used in the regression

For the quantitative bias analysis, participation fractions and observed data are listed in Table 4. In the naïve analysis comparing inner, outer and remote ARIA+ as one group to city, the estimated OR for death was 0.84 (95%CI: 0.59–1.20); however, in the deterministic sensitivity analysis this estimate reversed to OR = 1.83. The estimated percent bias was − 54%, which is a substantial. Similarly, when outer regional and remote were compared as a single group to inner regional and city as a single group the naïve estimate was OR = 0.69 (95%CI: 0.36–1.34), which was corrected to 1.59 when accounting for the participation fractions, with a similar estimate of bias of −54%.

**Table 4:** Participation fractions, observed data and estimated ORs for the quantitative bias analysis

**Model 1**		
*Participation fractions*	Exposed (Inner & outer regional, remote)	Unexposed (City)
Disease (died)	0.123	0.272
No Disease (alive)	0.931	0.948
*Observed data*		
Disease (died)	55	74
No Disease (alive)	1030	1165
*Estimated ORs for death*	OR	95% CI
Model	0.84	(0.59–1.20)
QBA	1.66	
**Model 2**		
*Participation fractions*	Exposed (Outer regional & remote)	Unexposed (Major city & inner regional)
Disease (died)	0.086	0.197
No Disease (alive)	0.941	0.940
*Observed data*		
Disease (died)	10	119
No Disease (alive)	237	1958
*Estimated ORs for death*	OR	95% CI
Model	0.69	(0.36–1.34)
QBA	1.59	

OR: odds ratio; 95% CI: 95% confidence interval; ref: reference level used in the regression

## Discussion

This study examined the effect of remoteness of injury on in-hospital mortality after major trauma in the State of Victoria for 2324 victims of Motor Vehicle Collisions admitted to the Royal Melbourne Hospital from 2010 to 2021. Just over half (53.3%) were injured in a major city, 36.1% were inner regional and 10.6% were in outer regional and remote regions. This corresponded to a slightly lower proportion of major city residents than the total Australian population (72% major City) [2]. Across the three remoteness categories examined, patients had a similar distribution of age, gender and comorbidity, but were dissimilar in terms of their injury severity and in-hospital mortality (Table 1).

We hypothesised that increasing remoteness of injury would be associated with an increased odds of in-hospital mortality. This was not observed in the data. To the contrary, the initial analysis suggested that there was a non statistically significant decrease in the adjusted odds of death for those injured in the most remote category (outer regional/remote) when compared to those injured in a major city. Our expectations were influenced by national statistics from the Australian Institute of Health and Welfare that suggested age adjusted rates of death and hospitalisation increase with remoteness of residence (2019-20 data, 41 per 10^5^ for those residing in Major Cities, 64 per 10^5^ for Outer Regional and 80 per 10^5^ for Very Remote) [1], as well as road safety data that suggested more road deaths occur in rural locations than urban locations [14]. It is noted that the results of the quantitative bias analysis were more compatible with the AIHW data,

However, various factors may have influenced this discrepancy. First, the national analysis examined remoteness of residence, not remoteness of injury, and is therefore relevant to persons living remotely, whereas our data are relevant to persons injured remotely. In our study, the correlation coefficient for ARIA+ category for injury location and home location was 0.70, representing a strong correlation, but not as high as anticipated given that ARIA+ categories are geographically very large. Perhaps it is inherent in transport-related injuries, that persons are more likely to be distant from their home address. Second, the overrepresentation of deaths in rural locations may be strongly influenced by the higher speeds allowed on rural roads, noting that risk of death doubles for every 5 km/hr traveling speeds over 60 km/h [14].

Third, our outcome of interest was in-hospital mortality, not in-hospital and out-of-hospital mortality, which was the outcome in the AIHW national analysis. Of the few studies that have specifically looked at in-hospital mortality, and hence are more methodologically comparable to our study, results have been similar the findings in our main analysis of no increase odds of death for those living in more remote areas. A study from Western Australia (OR = 1.10, 95% CI 0.66–1.84, *p* = 0.708 [15]) and two from Canada (OR 0.93, 95%CI 0.76–1.14 [16]; OR 0.93, 95% CI 0.58–1.46 [17]) all showed that mortality was no different once the patient reached a major trauma centre.

Restriction of the dataset to persons who survived to hospital limits the generalisability of our findings because those with a more fatal combination of injuries and comorbidities may be more likely to survive to hospital if injured in the more accessible (major cities) regions. However, we did not observe higher ISS scores among patients whose injuries occurred in the major city/inner regional categories, nor was comorbidity different across the ARIA+ categories, so other unmeasured factors may be contributing. Whilst this result was unexpected, other studies have suggested that a subset of rural trauma patients may have a survival advantage. A study of 11,000 major trauma patients from NSW also reported a potential survival advantage for those injured rurally who survived till hospital admission [9]. In that study, the rurally injured were half as likely to die in-hospital than persons injured in metropolitan areas [18]. A study of 30,000 major trauma patients for 24 Australian centres [5] also showed an inverse relationship between in-hospital mortality rates and increasing remoteness of injury, with 12.0% of those injured in major cities dying, 7.8% in inner/outer regional, and in 5.5% remote/very remote regions.

In our study, we examined whether this apparent inverse relationship might be associated with survivor bias using quantitative bias analysis [13]. Our corrected estimate suggested a 1.66-fold increased odds of death for more remotely injured persons. This result is more consistent with studies that have been able to include pre-hospital death in their analyses. For example, a study from Nebraska utilising population level data that included prehospital deaths reported a 1.98-fold increased odds of death for remote persons (95%CI 1.18–3.31) [6]. Similarly, increased odds of death for rurally-injured persons using regional registry data have been reported from Nova Scotia (OR = 1.66, 95%CI: [12]1.09–2.52 [17]), Alabama (4.2% remote vs. 2.2% urban, *p* = 0.001 [7]), and Quebec (OR = 3.44, 95% CI: 1.88 to 6.28) [16]. The largest study on the topic, a multicentre USA study of 8 million trauma patients, showed a more modest association between rural injury and death (OR = 1.14, 95%CI: 1.09–1.19; *p* < 0.001) [19], but that study used home rather than injury address. One Australian study from New South Wales showed an increased risk of in-hospital death (OR 1.75, 95% CI 1.06–2.89, *p* = 0.02) [18] for rurally injured persons.

In tandem, the results of our main analysis and the quantitative bias analysis suggest that, while rurally-injured patients are not more likely to die in hospital once admitted, a majority of deaths due to major motor vehicle trauma occur prior to any involvement of a major trauma centre. The finding that regional and remote patients do not have inferior survival compared to their city counterparts once they reach a major trauma centre is a useful quality assurance finding for a major trauma centre. However, the generalisability of our results, and those of other studies which also could not include prehospital deaths, is likely limited. Such studies should be interpreted with the understanding that the results apply to the subset of trauma victims who have survived to hospital. Any study which for which the goal is to estimate survival by remoteness of injury among all injured persons must include pre-hospital deaths.

We used the ARIA+ as our measure of remoteness. This was chosen over other measures, such as geographical region, straight line distance, time or receiving hospital trauma designation. It has been a long-held tenet of trauma care that reducing prehospital time will reduce mortality, but more recently this has come into question. A study from Sweden showed that ISS, penetrating mechanism, and age had more of an impact on mortality than prehospital time [20]. A recent systematic review similarly identified that prehospital time was not an influence on the odds of mortality for the majority of ‘undifferentiated’ trauma patients, the exception being the haemodynamically abnormal penetrating trauma victim and the patient with a serious head injury [21]. Population level data from a study from Norway [22] was subject to varying statistical models to explore the relationship between various prehospital factors and subsequent trauma mortality and concluded that the effect of pre-hospital time was minimal and the size of any effect was highly dependent upon the statistical model used. The strongest association with mortality was with decreasing population density of the site of injury, which could be construed as another measure of remoteness akin to ARIA+. A single Australian study on the topic was identified, similarly showed no association of pre-hospital time on 30-day mortality [23]. Our decision to not include prehospital time in our main analysis was not based on the studies cited above, but was a decision based on our assumed causal model in which prehospital time is shown as a mediator on the causal path between type of injury and death. Inclusion of prehospital time as a covariate in our regression analysis would therefore introduce bias [24]. To better understand the contribution of prehospital time to in-hospital death mediation analysis could be used [25] preferably using regional trauma registry data to avoid survivor bias. In addition, the mode of retrieval (e.g. helicopter, road ambulance) was not examined, which may have influenced pre-hospital time.

This was a single centre study with several limitations that may have harmed the interval validity of our findings. First, inclusion was conditioned upon survival. While the results are applicable to our hospital population, they may not be generalisable to the state. Moreover, we assessed the generalisability of our results through quantitative bias analysis and determined that our sample had limited external generalisability. A more comprehensive study on the topic would include prehospital deaths and deaths in intermediary hospitals. Nevertheless, the results provide reassuring evidence of no inequity of hospital care by location of injury. Second, ARIA+ category may have been inaccurately allocated. Whilst postcodes in the accessible regions usually contained 100% of the population in one ARIA+ category, more remote regions often had proportions of their populations straddling two ARIA+ categories. However, this is unlikely to have biased our results, as estimates were not appreciably changed when we used the second most-common ARIA+ category for postcodes with more than one category. Third, the important covariate of comorbidity was missing in 81 of the 2324 cases, which as a proportion was low, but there were important differences in the distribution of missingness by mortality outcome. We attempted to reduce the impact of missing comorbidity data through multiple imputation; however, the missingness was unlikely to have been completely at random. Restriction of this study to a single mechanism of injury was performed to achieve more comparable groups to better test the hypothesis, but this limits external validity to other mechanism of injury, particularly penetrating trauma which is believed to be a more time-sensitive disease. Finally, the findings of our study may not generalise to trauma systems in other geographic locations or even other hospitals within Victoria which may have different infrastructure available to support in-hospital care of trauma patients.”

Despite these limitations, this study provides new data on the relationships between remoteness of injury and trauma mortality in Victoria, Australia. It used ARIA as its measure of remoteness, which is arguably a more direct measure of access to services, compared to time and geographical subunit. Data quality was considered high, as data were collected contemporaneously by dedicated health information officers. This study also included adjustment for comorbidity age, a key covariate when examining survivability of a major trauma patient. Finally, the quantitative bias analysis has highlighted the importance of survivor bias when interpreting studies of remoteness of injury and trauma mortality.

## Conclusion

This study of 2324 adult persons suffering major trauma from a motor vehicle collision has not shown an increase in in-hospital mortality with increasing remoteness. Counter to expectations, in this dataset there was some evidence of a survival advantage for those injured in outer regional/remote areas who had half the odds of dying in hospital compared with those injured in a major city. However, this apparent survival advantage for more remotely injured persons is likely due to selection bias, as suggested in the quantitative bias analysis. Our findings are useful from a hospital quality assurance viewpoint, in that remote persons do not have inferior survival if they reach a major trauma centre. Future studies with data linkage from coronial and paramedic sources would allow further insights into survivability of remotely injured trauma patients from point of injury to pre-hospital or in-hospital death.

## Electronic supplementary material

Supplementary Material 1

## Data Availability

Data are available upon suitable ethical approval has been obtained. data is not publially avaialble as per the conitions of the Human Research Ethics committee approval.
