# Supplementary material for: The association between remoteness of injury and in-hospital mortality for motor vehicle collision major trauma patients: evidence of survivor bias in an analysis of registry data
Source: Inj Epidemiol. 2025 Jul 8;12:40. doi: 10.1186/s40621-025-00586-w (PMC12239381; doi:10.1186/s40621-025-00586-w)
Supplement: Supplementary file 1 — Supplementary Material 1 [file 40621_2025_586_MOESM1_ESM.docx]

## Supplementary Table 1. Comparison of key covariates between cases with missing and complete comorbidity data

|  |  | Missing comorbidity  n=81 | Complete comorbidity  n=2243 |
| --- | --- | --- | --- |
| Death | (%) | 44 (54.3) | 85 (3.8) |
| Prehospital hours, median (IQR) |  | 1.97 (1.7) | 1.95 (4.0) |
| Prehospital hours category | <1h | 11 (13.6) | 182 (8.1) |
|  | 1-6h | 64 (79.0) | 1529 (68.2) |
|  | >6 | 6 (7.4) | 532 (23.7) |
| Inpatient days, median (IQR) |  | 1.44 (13.2) | 7.9 (9.4) |
| ISS, median (IQR) |  | 30 (18) | 19 (12) |
| ISS>24 (%) |  | 57 (70.4) | 619 (27.6) |
| Age, years, median (IQR) |  | 37.5 (20.9) | 44.6 (21.1) |
| Age group | 15-24 | 32 (39.5) | 516 (23.0) |
|  | 24-44 | 25 (30.9) | 724 (32.3) |
|  | 45-64 | 13 (16.1) | 505 (22.5) |
|  | 65+ | 11 (13.6) | 498 (22.2) |
| Gender, (% female) |  | 26 (32.1) | 848 (37.8) |
| ARIA (%) | Major city | 41 (50.6) | 1200 (53.5) |
|  | Inner regional | 34 (41.9) | 803 (35.8) |
|  | Outer regional | 6 (7.5) | 233 (10.4) |
|  | Remote | 0 (0) | 6 (0.27) |
| Interhospital Transfer (% yes) |  | 5 (6.2) | 433 (19.3) |
| Prehospital transport | Road | 43 (55.8) | 1402 (68.2) |
|  | Air | 34 (44.2) | 647 (31.6) |
| Years | 2010 to median | 45 (55.6) | 1093 (48.7) |
|  | median to 2021 | 36 (44.4) | 1150 (51.3) |
| Major head injury n(%) | yes | 42 (51.9) | 574 (25.6) |
